# Supporting creative agency: metacognitive control and trust in generative AI art education

**DOI:** 10.3389/fpsyg.2026.1997631

**Published:** 2026-10-29

**Authors:** Renhao Zhu

**Affiliations:** Zhejiang Hengdian College of Film & Television, Dongyang, China

**Keywords:** art education, GenAI, metacognitive control, trust, UTAUT

## Abstract

This paper employs a quantitative multimethod design to extend the UTAUT framework for generative AI (GenAI) adoption among art students. Study 1 tested the integrated model via structural equation modeling using cross-sectional survey data from 450 Chinese art students and developed a trust construct grounded in instrumental technology trust, supplemented by art-value-aligned items. Trust in GenAI showed the strongest direct association with behavioral intention (*β* = 0.425). Social influence was the dominant correlate of trust (*β* = 0.262), with 42.1% of its total effect mediated indirectly through trust. Metacognitive control showed a positive direct association with behavioral intention (*β* = 0.146), alongside an indirect association through trust. The structural model based on survey data explained 59.7% of the variance in behavioral intention. Study 2 used a between-subjects experiment with 80 art students to test whether situational metacognitive prompting causally affected creative output. Participants receiving metacognitive prompts (Group A) obtained higher expert ratings than the control group (Group B) on semantic content (*d* = 0.67), visual style (*d* = 1.30), and spatial composition (*d* = 0.63); within-group feature-distance analyses also indicated greater output diversity in Group A across all three dimensions. These findings suggest that trust and metacognitive control are key pathways in GenAI adoption for artistic creation, and that low-cost situational metacognitive prompting may enhance both the quality and diversity of AI-assisted artworks.

## Introduction

Technological innovation drives educational reform. Generative AI (GenAI), which processes unstructured textual and visual data, has reshaped learning and knowledge production (Murugesan and Cherukuri, 2023). Consequently, scholars and practicing educators have begun to examine how GenAI tools reshape the logic of human learning, modes of knowledge production, and future career structures (Dwivedi et al., 2023). Although GenAI offers substantial educational benefits, the academic community should move beyond merely worrying about risks to teaching standards and instead explore reasonable, standardized guidelines for its use by students and scholars (Susarla et al., 2023). However, the adoption of emerging technologies is a gradual psychological process that poses a significant challenge for art students in higher education. Artistic creation itself relies on the personal styles and values that creators have developed over time. Students are generally concerned that integrating GenAI into the creative process may undermine their artistic autonomy. This concern has further slowed the adoption of GenAI. This paper adopts two complementary studies with distinct research aims. Study 1 used cross-sectional survey-based structural equation modeling to test the psychological adoption pathway: metacognitive control shapes art students’ behavioral intention to use GenAI both directly and indirectly via trust in GenAI. Study 2 employed complementary triangulation a between-subjects laboratory experiment to manipulate situational metacognitive prompts and examine their causal links to aesthetic quality and output diversity of AI-assisted artworks. The two studies adopt complementary triangulation. Study 2 does not test the trust-mediation pathway established in Study 1, as it focuses on creative-output outcomes instead of behavioral intention.

## Literature review

Most research on AI adoption in education still operates within the technology acceptance tradition. Studies in art and design education have largely extended these technology acceptance frameworks to identify the antecedents of students’ intention to use AI tools. This body of work has considerable value, but it rests on assumptions that are in tension with the actual process of artistic creation.

The first problem is conceptual: how “trust” is defined. In mainstream educational technology research, trust is treated as confidence in system stability and data security (Tarhini et al., 2017a). In artistic settings, students’ trust in AI is inseparable from normative judgments about authorship, the authenticity of the work, and aesthetic autonomy (Grassini and Koivisto, 2025). Acceptance models built on utilitarian beliefs have no vocabulary for this value-laden dimension, and so they tend to miss the very concerns that shape art students’ decisions.

The second is theoretical. Metacognitive control has largely been left out of the story: the literature has not explained how learners plan, monitor, and coordinate with AI during human–machine collaboration (Bian et al., 2025; Xu, 2025). The omission matters, because metacognitive control is central to creative ideation: task focus, goal-oriented strategy use, and effective creative screening each independently predict divergent creativity (Benedek and Lebuda, 2025). Without this pathway, acceptance models offer no account of how students actively regulate their cognitive processes while working with generative systems.

The third is empirical. Existing studies mostly verify main effects; they rarely trace how external technological conditions are converted into internal motivation through psychological mediating variables (Sáez-Velasco et al., 2024; Marqués Ibáñez and Delgado Hernández, 2025). GenAI can enhance mental flexibility and novelty by supplying divergent stimulus material (Tigre Moura et al., 2023), yet critics argue that reliance on algorithm-generated content fosters cognitive homogenization and weakens creative agency (Pan et al., 2025). The likely hinge between these outcomes is the user’s degree of control: minimal, directive prompts combined with passive acceptance tend to yield formulaic outputs, whereas active control and iterative refinement allow AI to amplify creativity (Sherson et al., 2023). Artistic creation, being open-ended and affectively charged, is precisely where this hinge matters most; because AI lacks intentionality and aesthetic sensibility, the human’s analytic and interpretive role remains irreplaceable (Wingström et al., 2024). Therefore, this study aims to extend the UTAUT-based technology acceptance model by examining whether art students’ GenAI use correlates with these new constructs, and by experimentally testing the causal effects of situational metacognitive prompting on the aesthetic quality and output diversity of AI-assisted artworks.

## Theoretical framework and research hypotheses

### Theoretical distinctiveness of GenAI-supported artistic creation

The Unified Theory of Acceptance and Use of Technology (UTAUT) is a widely used framework for studying information-technology user adoption. It helps researchers explain how users form attitudes toward and trust in GenAI and other emerging technologies (Venkatesh et al., 2003). Traditional UTAUT models aim to explain the adoption of information technology that functions primarily as a tool-based system. These constructs assume that adoption is largely a matter of functional evaluation and social conformity. For bounded, efficiency-oriented tasks, these assumptions are reasonable. However, for GenAI-supported artistic creation, art-generation processes have moved beyond simple linear task execution toward interactive, dialogue-like collaborative work. Beyond its core tool-related attributes of stability and reliability, GenAI also carries context-specific artistic considerations: originality, safety, and algorithmic fairness. In this context, the core issue of adoption is not merely whether GenAI is useful or easy to use, but whether it can be trusted as a legitimate medium for artistic creation.

GenAI-driven artistic creation is open-ended rather than bounded. This stems not only from its interactive nature during task work but also from differences between artistic and industrial evaluation standards. Artistic creation has no clear stopping criteria and thus demands ongoing monitoring, strategic adjustment, and iterative optimization. Metacognitive control has been theorized as an internal agency mechanism distinct from facilitating conditions. It directly influences behavioral intentions by providing strategic capabilities and a sense of authorial control, and indirectly influences behavioral intentions by reducing “black-box anxiety” and enhancing trust.

This study makes three modifications to the original UTAUT. First, use behavior was removed. The primary reason was that the survey’s inclusion criteria restricted the sample to art students who had already used GenAI in their disciplinary studies. As a result, “whether to use” was largely predetermined, and a use-behavior measure would suffer from restricted variance, selection bias, and ceiling effects, substantially weakening its appropriateness and explanatory power. A secondary reason is that self-reported Use behavior in cross-sectional data is vulnerable to common method bias, social desirability, and reverse causality; moreover, GenAI use is highly heterogeneous, ranging from casual exploration to iterative creation, making it difficult to quantify uniformly by frequency or time. Therefore, Study 2 provided complementary behavioral evidence by experimentally examining creative output diversity.

Second, facilitating conditions were modeled as antecedents of behavioral intention rather than of use behavior. Because this study does not adopt use behavior as the dependent variable, the original UTAUT path from FC to use behavior was no longer applicable. Subsequent extensions suggest that in early-stage educational technology adoption, external resources and support are better understood as enabling conditions that shape users’ willingness to adopt. In GenAI-supported artistic creation, the availability of hardware, training, and assistance does not automatically produce creative use; it first increases perceived manageability of the creative process and reduces uncertainty and risk, thereby strengthening trust in GenAI and directly enhancing behavioral intention. We therefore model FC → BI (H3) and FC → Trust (H7) to capture how external conditions were converted into behavioral intention and value-laden trust, rather than directly producing use behavior.

Third, regarding the newly added GenAI trust construct, this study acknowledges that it partially overlaps with traditional technology trust. However, this overlap was not a theoretical flaw; rather, it represents a reasonable extension and contextualization. The scale was adapted from an established technology trust scale to the artistic context and refined in a pilot study. This pilot study (*N* = 121) was conducted prior to the main survey, using the same recruitment method as the main survey: participants were art majors who met the same inclusion criteria, and the study was distributed through the network of teachers in Zhejiang. It is important to note that the pilot study sample was independent of the 450 participants in Study 1 and is not included in that study. During the pilot, the trust scale initially included two authorship-related items (BHA1 and BHA3) in addition to the five retained trust items (TI1–TI5). An exploratory factor analysis (EFA) with principal axis factoring and varimax rotation revealed a clear two-factor structure: the five trust items loaded on the first factor (loadings 0.714–0.788), whereas the two authorship items loaded cleanly on a separate second factor (BHA3 = 0.845; BHA1 = 0.773) with no cross-loadings on the trust factor (Supplementary Table S3). Because these two items formed a distinct factor that was not theoretically consistent with a unidimensional trust construct, they were removed at the EFA stage to preserve a single-factor trust scale for the CFA.

Within the final five-item scale, TI1 (functional reliability), TI3 (safety and privacy), and TI5 (overall reliability) overlapped with traditional technology trust, and this overlap constituted the foundational core of trust: students must first believe that the system can operate stably and protect their works and data. However, foundational trust alone is insufficient to explain adoption decisions in the artistic context, because students also care about whether generated content is original, whether authorship is respected, and whether the algorithm is fair. Therefore, TI2 (originality and authorship) and TI4 (algorithmic fairness) reflected the value-laden dimensions specific to artistic creation. Because only two of the five retained items capture art-specific concerns, we described the construct as primarily instrumental trust with an art-value supplement.

### Variables under the unified theory of acceptance and use of technology (UTAUT)

This study draws on theoretical extensions in subsequent research on technology acceptance to treat facilitating conditions as direct antecedents influencing behavioral intention. Chen et al. (2026) investigated older adult learners’ acceptance of generative AI and reported a substantial, statistically significant positive effect of facilitating conditions on behavioral intention (*β* = 0.351, *p* < 0.05), where FC acted as the second-strongest predictor of BI among UTAUT core variables. Xu and Zhang (2026), targeting Chinese university students’ academic use of DeepSeek, also validated a significant direct FC-to-BI relationship (*β* = 0.081, *p* < 0.05), albeit with a comparatively smaller effect size. We hypothesized that when students perceive sufficient hardware, learning resources, technical support, and assistance channels, their intention to use GenAI will significantly increase. Based on the above theoretical framework, the following hypotheses are proposed (Figure 1):

**Figure 1:**
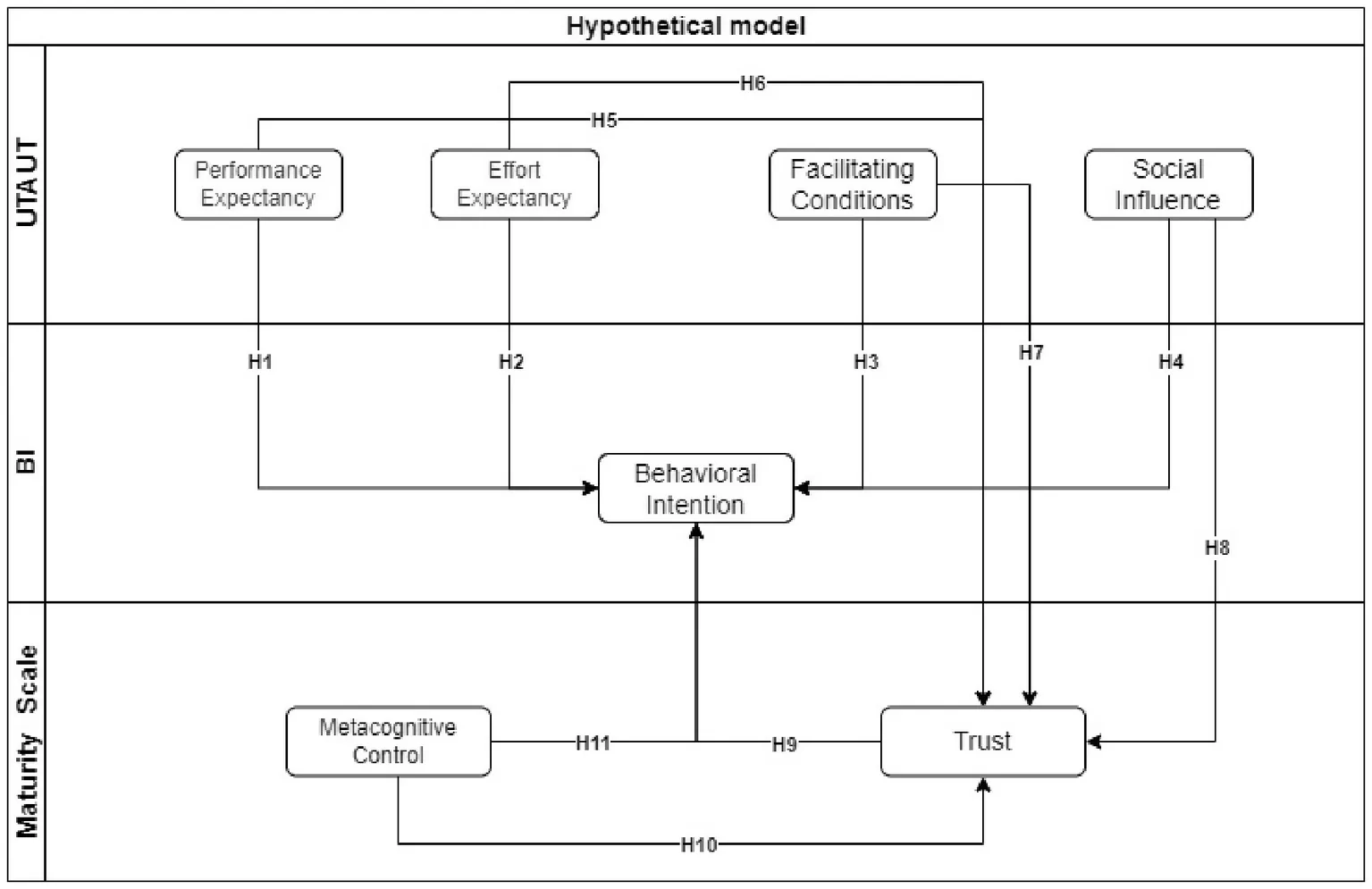
Hypothesized research model.

*H1*: Performance expectancy positively influences art students’ behavioral intention.

*H2*: Effort expectancy positively influences art students’ behavioral intention.

*H3*: Facilitating conditions positively influence art students’ behavioral intention.

*H4*: Social influence positively influences art students’ behavioral intention.

### Extended variables: trust and metacognitive control

In this study, trust in GenAI is defined as art students’ confidence in GenAI under conditions of uncertainty and risk regarding creative needs, originality, safety, fairness, and reliability. Students’ concerns extend far beyond functional efficiency at the tool level; they delve deeper into value-based issues such as the attribution of originality in AI-generated content, the potential risk of copyright infringement of existing works, and whether their artistic identity and creative autonomy are being eroded by algorithms (Ansone et al., 2025; Wu et al., 2026). Precisely because of these unique concerns, trust plays a far more significant role in GenAI adoption decisions than in traditional information systems and is regarded by most scholars as the core psychological mechanism linking technological attributes to user behavior (Zhou et al., 2024; Wang et al., 2025).

*H5*: Performance expectancy positively influences art major students’ trust in GenAI.

*H6*: Effort expectancy positively influences art major students’ trust in GenAI.

*H7*: Facilitating conditions positively influence art major students’ trust in GenAI.

*H8*: Social influence positively influences art major students’ trust in GenAI.

*H9*: Trust in GenAI positively influences art major students’ behavioral intention.

Metacognitive control refers to an individual’s capacity to strategically plan, continuously monitor, and flexibly adjust cognitive strategies while collaborating with GenAI on academic or creative-production tasks (Mahy and Li, 2026). This construct originates from the theoretical framework of self-regulated learning in cognitive psychology and includes four core dimensions (Mahy and Li, 2026):

1. Planning: Prior to using GenAI, students formulate targeted interaction strategies according to task characteristics.
2. Monitoring: During human–GenAI collaboration, students constantly evaluate whether AI outputs support the achievement of task goals.
3. Regulation: In response to real-time feedback, students flexibly adjust interaction patterns to improve collaborative creative results.
4. Role Awareness: Students explicitly clarify the division of responsibilities and boundary definitions between human creators and AI in collaborative creation.

The reason artistic creation requires more metacognitive control than information retrieval or data processing is not primarily because the tasks are “more difficult,” but because the evaluation criteria are more ambiguous, the stopping rules are less clear, and strategy shifts occur more frequently. Creative tasks are typically ill-defined and have no single correct answer; therefore, creators must continuously monitor their progress, adjust their strategies, and decide which ideas to retain, develop, or discard (Lebuda and Benedek, 2023). This means that creators must distinguish between the problem space and the solution space, and adjust their process according to the stage they are in; skipping a stage requires metacognitive reflection to identify and correct the issue (Von Thienen et al., 2023). Based on the above theoretical framework, the following hypotheses are proposed:

*H10*: Metacognitive control positively influences art major students’ Trust in GenAI.

*H11*: Metacognitive control positively influences art major students’ behavioral intention.

## Materials and methods

### Study 1: survey study

#### Questionnaire design

This study employed a self-administered structured questionnaire that underwent multi-stage validation to ensure psychometric robustness and situational appropriateness. The questionnaire consisted of two parts, and all items measuring latent variables were adapted from established scales in the field of technology acceptance.

To ensure cross-linguistic equivalence, a standardized forward-backward translation procedure was adopted (Guillemin et al., 1993). Demographic questions and latent-variable measures appeared in separate survey modules to create psychological distance between predictor and outcome measures. An anonymity statement was prominently placed at the beginning of the questionnaire to reduce social desirability bias (Tehseen et al., 2017); all items were phrased in a neutral and concise manner, and the scale uniformly used a 5-point Likert scale ranging from 1 (strongly disagree) to 5 (strongly agree).

The first section of the questionnaire collected four demographic characteristics: gender, major, educational attainment, and self-rated computer proficiency (Appendix Table A1); the second section measured seven core constructs comprising a total of 26 items, all adapted from well-validated scales and tailored to the context of artistic creation (Appendix Table A2) (Liu et al., 2015; Tarhini et al., 2017b; Birch and Irvine, 2009; Wrycza et al., 2017; Yao et al., 2022; Venkatesh et al., 2003; Zhu et al., 2025; Venkatesh et al., 2003; Cao et al., 2021; Mahy and Li, 2026; Chai et al., 2021).

#### Data collection

This study adopted a purposive sampling strategy, recruiting participants through an online survey. The inclusion criteria were as follows: enrollment in a regular higher education institution in Zhejiang Province, China; current student status in an art-and-design-related major; and prior experience in using generative artificial intelligence technology within their disciplinary studies.

Data were collected via the online questionnaire platform “Wenjuanxing” from April 1 to April 29, 2026. Questionnaire links were distributed by faculty collaborators at eight higher education institutions across Zhejiang Province, China, to students who met the inclusion criteria. After retrieval, rigorous data cleaning was performed: five invalid questionnaires were removed for excessively short completion time, identical responses across all items, or apparent logical contradictions. Consequently, 450 valid questionnaires were obtained, yielding a valid response rate of 98.9%.

#### Reliability and validity analysis

Internal consistency reliability of the measurement instrument was evaluated using Cronbach’s alpha coefficient, the most widely used indicator for assessing the homogeneity of scale items. As presented in Table 1, the overall Cronbach’s alpha for the entire questionnaire reached 0.927 (George and Mallery, 1999), indicating excellent internal consistency at the scale level. For the seven latent constructs, Cronbach’s alpha values ranged from 0.874 to 0.909. All construct-level alpha coefficients exceeded the conventional threshold of 0.70 for acceptable reliability, and the majority surpassed 0.80, demonstrating good to excellent internal consistency for each individual dimension.

**Table 1:** Descriptive statistics and details of measurement items.

Factor and item	Mean	SD	Skewness	Kurtosis	Cronbach’s alpha
Performance expectancy					0.882
PE1	3.19	1.032	−0.099	−0.518	
PE2	3.32	1.008	−0.056	−0.392	
PE3	3.3	1.01	−0.149	−0.521	
Effort expectancy					0.892
EE1	3.28	0.994	−0.253	−0.319	
EE2	3.29	1.099	−0.141	−0.72	
EE3	3.53	1.133	−0.277	−0.836	
Facilitating conditions					0.887
FC1	3.1	1.018	−0.108	−0.462	
FC2	3.7	1.156	−0.591	−0.459	
FC3	3.4	0.981	−0.307	−0.169	
FC4	3.34	1.104	−0.304	−0.523	
Social influence					0.909
SI1	3.34	1.049	−0.259	−0.396	
SI2	3.18	1.075	−0.071	−0.636	
SI3	2.89	1.014	0.035	−0.362	
Metacognitive control					0.874
MC1	3.1	0.976	0.063	−0.463	
MC2	3.39	0.954	−0.206	−0.157	
MC3	3.32	0.974	−0.075	−0.437	
MC4	3.11	0.951	−0.068	−0.284	
Trust in GenAI					0.902
TI1	3.25	1.058	−0.096	−0.567	
TI2	3.19	1.011	−0.087	−0.457	
TI3	3.26	1.032	−0.147	−0.581	
TI4	3.15	1.034	−0.018	−0.533	
TI5	3.24	1.016	−0.144	−0.431	
Behavioral intention					0.905
BI1	3.32	1.14	−0.188	−0.74	
BI2	3.13	1.094	−0.069	−0.626	
BI3	3.74	1.214	−0.623	−0.543	
BI4	3.15	1.094	−0.083	−0.577	
Overall questionnaire					0.927

SD = standard deviation.

Descriptive statistics for all items are also reported in Table 1. The mean scores of the items ranged from 2.89 (SI3) to 3.74 (BI3), with standard deviations between 0.951 (MC4) and 1.214 (BI3). The absolute values of skewness and kurtosis for all items were less than 1, suggesting that the data approximately followed a normal distribution.

As illustrated in Table 2, all standardized item loadings spanned from 0.765 to 0.894, uniformly surpassing the 0.6 threshold indicative of robust item-to-construct correspondence. Reliability metrics further attested to satisfactory internal consistency: composite reliability (CR) estimates for all latent constructs ranged between 0.875 and 0.909, comfortably exceeding the 0.7 benchmark. Meanwhile, average variance extracted (AVE) values varied from 0.636 to 0.770, each exceeding the conventional cutoff of 0.5, which collectively substantiates strong convergent validity across all unidimensional latent constructs.

**Table 2:** Confirmatory factor analysis of the variables.

Factor	Item	Standardized factor loading	CR	AVE
Performance expectancy	PE3	0.865	0.883	0.715
	PE2	0.834		
	PE1	0.837		
Effort expectancy	EE3	0.86	0.894	0.737
	EE2	0.861		
	EE1	0.854		
Facilitating conditions	FC4	0.816	0.889	0.666
	FC3	0.798		
	FC2	0.806		
	FC1	0.844		
Social influence	SI1	0.851	0.909	0.770
	SI2	0.887		
	SI3	0.894		
Metacognitive control	MC1	0.775	0.875	0.636
	MC2	0.765		
	MC3	0.801		
	MC4	0.846		
Trust in GenAI	TI1	0.789	0.903	0.649
	TI2	0.829		
	TI3	0.804		
	TI4	0.804		
	TI5	0.803		
Behavioral intention	BI4	0.824	0.906	0.707
	BI3	0.818		
	BI2	0.856		
	BI1	0.864		

CR = composite reliability; AVE = average variance extracted.

Discriminant validity was triangulated using two complementary rigorous methodologies to mitigate single-test bias. First, the Fornell–Larcker test results are presented in Table 3, wherein diagonal cells correspond to the square root of each construct’s AVE and off-diagonal cells represent inter-construct correlation coefficients. In compliance with the Fornell–Larcker axiom, the square root of AVE for every latent factor was greater than all bivariate correlation coefficients between the focal construct and its exogenous counterparts within the respective row and column (Fornell and Larcker, 1981).

**Table 3:** Fornell–Larcker test for discriminant validity.

Factor	PE	EE	FC	SI	MC	TI	BI
PE	**0.846**						
EE	0.356	**0.858**					
FC	0.352	0.31	**0.816**				
SI	0.17	0.286	0.316	**0.877**			
MC	0.311	0.338	0.306	0.273	**0.797**		
TI	0.427	0.452	0.466	0.451	0.369	**0.806**	
BI	0.446	0.471	0.471	0.467	0.455	0.699	**0.841**

Bold diagonal values are the square roots of AVE. Off-diagonal entries are inter-construct correlations.

Second, the HTMT ratio was deployed as a more conservative supplementary discriminant validity check (Table 4). All pairwise HTMT coefficients fluctuated within the interval [0.168, 0.699], with the maximum magnitude (0.699 for the BI–TI pairing) remaining substantially beneath the stringent 0.85 threshold. Congruent outcomes from both the Fornell–Larcker and HTMT tests jointly established that each latent construct possessed sufficient empirical distinctiveness, thereby confirming satisfactory discriminant validity of the measurement instrument (Henseler et al., 2014).

**Table 4:** Heterotrait–monotrait ratio of correlations (HTMT) values.

Factor	PE	EE	FC	SI	MC	TI	BI
PE							
EE	0.352						
FC	0.357	0.311					
SI	0.168	0.285	0.317				
MC	0.310	0.333	0.307	0.272			
TI	0.426	0.450	0.469	0.449	0.371		
BI	0.445	0.470	0.473	0.468	0.461	0.699	

#### Model fit index

A series of global fit statistics were computed to evaluate the empirical fitness of the reflective measurement model, common method factor model, and structural equation model, as tabulated in Table 5.

**Table 5:** Model fit indices.

Model fit indices	Threshold	Measurement model	Common method factor model	Difference (Δ)	Structural SEM model
CMIN		413.413	362.794	50.619	530.287
DF		278	252	26	384
CMIN/DF	<3	1.487	1.440	0.047	1.381
CFI	>0.9	0.982	0.985	0.003	0.981
GFI	>0.8	0.937	0.944	0.007	0.931
AGFI	>0.8	0.920	0.922	0.002	0.916
IFI	>0.9	0.982	0.986	0.004	0.981
TLI	>0.9	0.979	0.981	0.002	0.978
RMSEA	<0.08	0.033	0.031	0.002	0.029
NFI	>0.9	0.948	0.954	0.006	0.934
RMR	<0.05	0.030	0.026	0.004	0.032
SRMR	<0.08	0.027	0.024	0.003	0.0325

For the baseline measurement model, all fit statistics met the recommended criteria, indicating that the hypothesized measurement structure adequately fitted the collected empirical data. To diagnose potential common method bias (CMB) arising from single-source, self-reported survey data, we adopted the unmeasured latent common method factor (CLF) technique. Specifically, every observed indicator was specified to load on the CLF in addition to its substantive latent construct, the CLF was specified as orthogonal to the substantive factors, and no additional cross-loadings among substantive constructs were imposed. For model identification, the variance of the CLF was fixed to 1, while all loadings on it were freely estimated. The differences between the baseline measurement model and the common method factor model were negligible across all core fit indicators (ΔCMIN/DF = 0.047, ΔCFI = 0.003, ΔTLI = 0.002, ΔRMSEA = 0.002). Because the CLF accounted for little additional variance, the model fit improved only marginally, indicating no evidence of serious common method bias. As a cross-validation, Harman’s single-factor test was also conducted on all 26 scale items: the first unrotated factor explained 35.89% of the total variance, well below the conventional 50% threshold, further corroborating that common method bias is unlikely to be a serious concern in this dataset.

### Study 2: experimental study

#### Rationale and research objectives

Study 2 used a between-subjects experiment to test whether the situational component of metacognitive control causally affect creative output. Study 1 established that MC is directly associated with behavioral intention and indirectly associated through trust. Study 2 does not test this mediation path; trust was not measured. Instead, it isolates the manipulable, situational component of MC, namely strategy prompts and scaffolds during AI-assisted creation, and examines its causal effect on creative quality and diversity. Study 2 thus supplements the MC to BI direct path identified in Study 1 with preliminary causal evidence, while operating at a different level: output quality rather than behavioral intention. The two studies use complementary triangulation. Together, they converge on the central role of metacognitive control in creative engagement, although they do so through different methods and at different outcome levels. Participants were recruited for homogeneous baseline characteristics and completed a standardized AI-generation task. Creative expression was decomposed into semantic content, visual style, and spatial composition.

#### Participants

All 80 participants were recruited from the valid sample of 450 art majors in Study 1. Study 1 respondents who met the inclusion criteria and consented to follow-up contact were invited to sign up voluntarily for a laboratory session, and enrollment was completed within the fixed 2-day window. Participants were then assigned to the metacognitive-control group (Group A; *n* = 40) or the control group (Group B; *n* = 40) by simple random assignment at a 1:1 ratio using a random number generator. Among them, 32 were male and 48 were female, aged 18–24 years (*M* = 20.35, SD = 1.27). All participants had normal or corrected-to-normal vision, had prior basic experience in using GenAI tools, and signed informed consent before the experiment. Baseline equivalence was verified across all baseline variables: baseline metacognitive-control score [*t*(78) = 0.18, *p* = 0.858], AI use experience (Mann–Whitney *U* = 788.00, *p* = 0.903), age [*t*(78) = 0.84, *p* = 0.405], and sex [χ^2^(1) = 0.00, *p* = 1.000]; no baseline variable differed significantly between groups (all *p* > 0.05). Because random assignment occurred after recruitment, self-selection is unlikely to have systematically biased the between-group comparison, although this cannot be entirely ruled out. At the same time, the voluntary nature of participation means that the sample may not fully represent the broader Study 1 population, which limits the external generalizability of the findings.

All participants used the same text-to-image generation model, Nano Banana 2 (official model name: Gemini 3.1 Flash Image), developed by Google DeepMind and released on February 26, 2026. The model was accessed through the Gemini app using the model identifier gemini-3.1-flash-image-preview. Because Nano Banana 2 is a preview model, it may be updated by the provider without notice. To ensure version stability, all image generation was performed in batches within a fixed two-day window (May 3–4, 2026). The two groups were distributed evenly across the 2 days (Day 1: 20 experimental and 21 control participants; Day 2: 20 experimental and 19 control), and the order of conditions was alternated so that no batch was composed of a single condition. Every batch used the identical model identifier and interface version, the same fixed output parameters (resolution 1 K, 1:1 aspect ratio, 15-min task window), and no custom fine-tuning, plug-ins, or parameter presets. The interface version and model identifier were recorded at the start of each session, and no model updates or interface changes were observed during the study period.

Nano Banana 2 was selected for three reasons. First, it offers high-fidelity image generation with strong instruction-following capability, which is essential for a standardized task in which all participants must generate an image based on the same keywords. Second, it supports high subject consistency and precise text rendering, reducing the likelihood that group differences are confounded by model failures or refusals. Third, it is freely available to Gemini users and provides fast generation speed, making it feasible for participants to complete multiple generation attempts within the 15-min window. Its improved natural language understanding allows the model to capture subtle differences in participants’ prompts, which is critical for detecting metacognitive-control effects on creative outcomes.

#### Experimental design and procedure

This study employed a one-factor between-subjects experimental design in which the independent variable was the presence versus absence of metacognitive control. The primary dependent variable was expert aesthetic evaluation of the submitted AI-assisted artworks across three dimensions: semantic content, visual style, and spatial composition. The secondary dependent variable was the within-group pairwise feature distance in the same three dimensions, computed using open-source models as an objective, auxiliary indicator of creative homogeneity. Participants were randomly assigned to the experimental group or the control group at a 1:1 ratio. Participants were kept blind to condition assignment, and all expert raters were blinded to group assignment. All experimental procedures were conducted in accordance with standardized laboratory protocols. The experiment was divided into four consecutive phases. In the baseline confirmation phase, participant identity was verified, informed consent was obtained, and standardized task instructions were provided. In the manipulation phase, participants in the experimental group received four-stage metacognitive prompts (Supplementary Table S1) prior to creation and were instructed to record their revised prompts afterward, whereas participants in the control group received only the standard task instructions and no metacognitive prompts. In the standardized AI-assisted creation task phase, all participants were required to use the Nano Banana 2 model with fixed parameters, an output resolution of 1K, and a 1:1 aspect ratio to generate one image within 15 min based on the keywords “balcony” and “cat,” and to submit one final AI-assisted artwork. In the outcome assessment phase, each submitted artwork was evaluated by three blinded experts and by machine-based feature-distance analysis.

The keywords “balcony” and “cat” were selected *a priori* for four reasons. First, they form a natural scene–subject combination: “balcony” specifies the setting, whereas “cat” specifies the main subject. This combination allows meaningful variation in semantic content, visual style, and spatial composition while keeping the task sufficiently constrained for a 15-min standardized creation task. Second, both words are concrete, familiar, and highly imageable, which reduces the influence of prior domain knowledge and vocabulary differences across participants. Prior research has established that imageability is positively correlated with the effectiveness of verbal cues in creative tasks (Johnson and Hass, 2022). Third, the combination is open-ended enough to permit diverse stylistic and compositional solutions (Oppenlaender, 2022), including realistic, illustrative, cinematic, minimalist, or fantasy approaches, as well as different camera angles, color palettes, and lighting conditions. Fourth, the keywords are neutral with respect to culture, profession, and sensitive content, and they avoid copyright or safety concerns that could trigger model refusals or filtering.

#### Expert aesthetic evaluation procedure

Expert aesthetic evaluation served as the primary outcome measure in Study 2. Three expert reviewers, blinded to group assignment, independently evaluated 80 AI-assisted artworks. Each artwork was scored on a 7-point Likert-type scale across three dimensions—semantic content, visual style, and spatial composition—according to the behavioral anchors specified in the Expert Review Form (Supplementary Documents). Reviewers were required to carefully read the review instructions and scoring criteria prior to evaluation to ensure consistent interpretation of the dimensions and anchors. Reviewers completed the ratings independently, were instructed not to discuss their scores, and were asked to base all judgments solely on direct observation of the artworks, without inferring author identity, creative process, or generation method. The expert rating scale was designed to assess aesthetic quality across semantic content, visual style, and spatial composition. It does not directly measure creativity or creative ability.

For each artwork, dimension scores were averaged across the three experts, and an overall aesthetic score was computed as the unweighted mean of all nine ratings. Inter-rater reliability was assessed using ICC(2,k) or Cronbach’s alpha. Group differences were tested using independent-samples *t*-tests with Welch correction, and Cohen’s *d* was reported as the effect size. Multiple comparisons across the three dimensions and the overall score were corrected using the Benjamini–Hochberg false discovery rate procedure. All tests were two-tailed with α = 0.05. Accordingly, the study conclusions were derived from blinded expert aesthetic evaluations as the primary evidence, with machine-based feature distances serving only as a secondary, objective validation.

#### Machine-based feature extraction and comparison

All feature extraction and quantitative comparison procedures were implemented via self-developed offline Python scripts to eliminate subjective evaluation bias, with all pre-trained models loaded locally without online fine-tuning to guarantee reproducibility. To provide an objective, secondary validation of creative homogeneity, image features were extracted along three dimensions using open-source models. Semantic content representations were derived from an OpenCLIP ViT-B/32 image encoder, yielding a 512-dimensional embedding for each image. These embedding were L2-normalized, and pairwise content distance was operationalized as cosine distance, defined as 1 minus the cosine similarity between normalized embedding (Zhu et al., 2024). Visual style representations were obtained from an ImageNet-pretrained VGG19 network. Feature maps at indices 4, 9, 18, 27, and 36 of the torchvision VGG19 features module were used to compute layer-specific Gram matrices, which were flattened and concatenated into a single style vector. Pairwise style distances were computed using Euclidean distance (Sun et al., 2017). Spatial composition representations were derived from histogram of oriented gradients (HOG) descriptors. Each image was converted to grayscale, resized to 256 × 256 pixels, and represented by HOG features with 9 orientations, 32 × 32 pixels per cell, and 2 × 2 cells per block. Pairwise composition distances were calculated using Euclidean distance (Talaoubrid et al., 2022).

For each dimension (Table 6), a complete pairwise distance matrix was constructed across all images. Within-group mean distances were computed separately for the experimental group (A) and the control group (B), and the test statistic was defined as the difference between these means (A − B). Statistical significance was assessed using a strict permutation test with 10,000 permutations. Under the null hypothesis of no group effect, the group labels are exchangeable because participants were randomly assigned to the metacognitive prompting condition or the control condition, and the standardized task and model settings ensure that the generated images are otherwise comparable across groups. In each permutation, group labels were randomly reassigned while preserving the original group sizes, and the test statistic was recomputed, thereby maintaining the dependency structure of the full distance matrix. The two-sided *p*-value was computed as (b + 1) / (N + 1), where b denotes the number of permuted statistics with absolute values greater than or equal to the absolute observed statistic and *N* = 10,000. Bootstrap resampling with 10,000 replicates was performed within each group to estimate 95% confidence intervals for the difference using the percentile method. The permutation test provides a distribution-free *p*-value for the null hypothesis of no group difference, whereas the bootstrap confidence interval quantifies the uncertainty of the effect size estimate. Reporting both allows us to separate statistical significance from the precision of the estimated difference. Multiple comparisons across the three dimensions were corrected using the Benjamini–Hochberg false discovery rate procedure. All analyses were implemented in Python with a fixed random seed of 42 to ensure reproducibility. Larger within-group distances indicate greater heterogeneity and, consequently, lower creative homogeneity. However, several objective limitations of the feature-distance metrics described above warrant caution. First, CLIP embedding, VGG19 Gram matrices, and HOG descriptors capture specific statistical properties—such as semantic embedding, texture statistics, and edge-gradient structures—and cannot fully represent the aesthetic and semantic richness of artistic content, style, and composition. Second, the within-group mean distance reflects the dispersion or heterogeneity of images in feature space and is not equivalent to creativity, creative quality, or aesthetic value. A larger distance may arise from greater randomness, generation noise, model artifacts, or stylistic drift rather than from more creative expression. Accordingly, these metrics serve only as an auxiliary objective validation of group differences in homogeneity; they cannot replace expert judgments of aesthetic quality and should not be used alone to infer that the experimental group is superior in creativity.

**Table 6:** Feature representations and distance definitions across the three dimensions.

Dimension	Feature extraction method	Feature representation	Distance metric	Interpretation of distance
Semantic content	CLIP encode_image, L2-normalized	CLIP image embedding vector	Cosine distance	Semantic content similarity; smaller values indicate greater similarity.
Visual style	Gram matrices from VGG19 layers 4, 9, 18, 27, and 36	Concatenated multi-layer Gram vectors	Euclidean distance via cdist	Similarity in texture, brushwork, and style statistics.
Spatial composition	HOG: grayscale, resized to 256 × 256, 9 orientations, 32 × 32 pixels per cell, and 2 × 2 cells per block	HOG feature vector	Euclidean distance via cdist	Similarity in edges, gradients, and compositional structure.

Distance values in this table refer to pairwise distances between images within each dimension’s feature space: for a given pair of images, a smaller distance indicates greater similarity. For group-level comparisons, the within-group mean distance is computed by averaging all pairwise distances among images in the same experimental group, and a larger within-group mean indicates greater heterogeneity within that group. Because the three dimensions use different feature representations and distance metrics, distance values are not directly comparable across dimensions.

## Hypotheses results

### SEM results

As shown in Table 7, all 11 hypotheses were supported. Regarding the predictors of trust in GenAI, the results indicated significant standardized coefficients for performance expectancy (*β* = 0.202, *p* < 0.001), effort expectancy (*β* = 0.204, *p* < 0.001), facilitating conditions (*β* = 0.219, *p* < 0.001), social influence (*β* = 0.262, *p* < 0.001), and metacognitive control (*β* = 0.099, *p* = 0.042), all with *p*-values < 0.05, supporting Hypotheses H5, H6, H7, H8, and H10 (Figure 2).

**Table 7:** Hypothesis testing.

Hypothesis	Variable	Path	Independent variable	Un. estimate	S.E.	C.R.	*P*	Estimate	Result
H5	TI	←	PE	0.194	0.047	4.108	***	0.202	Accepted
H6			EE	0.175	0.042	4.153	***	0.204	Accepted
H7			FC	0.202	0.046	4.373	***	0.219	Accepted
H10			MC	0.109	0.053	2.035	0.042	0.099	Accepted
H8			SI	0.246	0.044	5.545	***	0.262	Accepted
H1	BI	←	PE	0.132	0.046	2.859	0.004	0.128	Accepted
H2			EE	0.105	0.041	2.555	0.011	0.113	Accepted
H3			FC	0.092	0.045	2.05	0.04	0.092	Accepted
H9			TI	0.459	0.059	7.727	***	0.425	Accepted
H11			MC	0.174	0.052	3.379	***	0.146	Accepted
H4			SI	0.157	0.043	3.611	***	0.155	Accepted
Demographic information			Gender	−0.044	0.063	−0.699	0.485	−0.024	Rejected
			Major	0.041	0.031	1.301	0.193	0.045	Rejected
			Computer	0.002	0.036	0.057	0.955	0.002	Rejected
			Education	0.073	0.036	2.04	0.041	0.072	Accepted

***p < 0.001.

**Figure 2:**
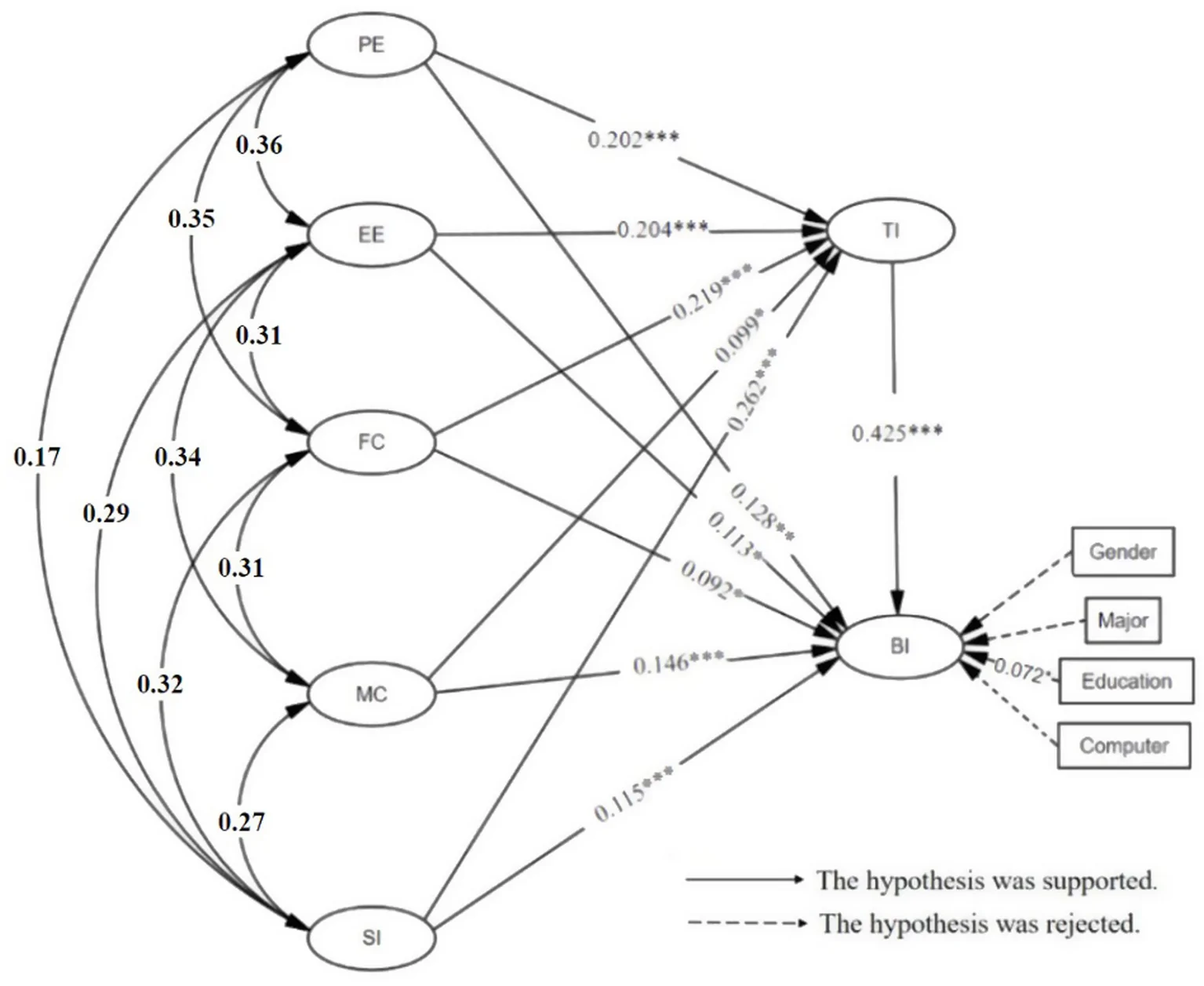
Structural model of impact pathways. *p < 0.05, **p < 0.01, ***p < 0.001.

Regarding the predictors of behavioral intention, the standardized coefficients for performance expectancy (*β* = 0.128, *p* = 0.004), effort expectancy (*β* = 0.113, *p* = 0.011), facilitating conditions (*β* = 0.092, *p* = 0.040), social influence (*β* = 0.155, *p* < 0.001), trust in GenAI (*β* = 0.425, *p* < 0.001), and metacognitive control (*β* = 0.146, *p* < 0.001) were all significant (*p* < 0.05), confirming Hypotheses H1, H2, H3, H4, H9, and H11. The structural model accounted for 43.5% of the variance in trust in GenAI (*R*^2^ = 0.435) and 59.7% of the variance in behavioral intention (*R*^2^ = 0.597), indicating substantial explanatory power of the hypothesized model.

This study also examined the influence of control variables (gender, major, computer proficiency, and education) on behavioral intention. The results indicated that gender, major, and self-rated computer proficiency had no significant effect on intention (all *p* > 0.05), whereas educational attainment exhibited a significant positive impact (*β* = 0.072, *p* = 0.041).

Bootstrap mediation analysis with 10,000 resamples was conducted to examine the mediating role of trust (Table 8) (Bollen and Stine, 1990). The results revealed significant indirect effects via trust for all five antecedents, as the bias-corrected 95% confidence intervals excluded zero. Partial mediation was established for performance expectancy, effort expectancy, social influence, metacognitive control, and facilitating conditions, given that their direct effects on behavioral intention remained significant after incorporating the mediator. For facilitating conditions, the direct effect was small but significant (*β* = 0.092, *p* = 0.040, 95% CI [0.003, 0.179]), indicating partial rather than full mediation through trust. Because this direct effect was close to the significance threshold, the partial mediation pattern should be interpreted cautiously (Preacher and Hayes, 2008).

**Table 8:** Results of bootstrap mediation analysis.

Path	Effect type	Point estimate	Bootstrapping						Mediation type
			Coefficient multiplication		Bias-corrected 95% CI		Percentile 95% CI		
			SE	Z	Lower	Upper	Lower	Upper	
PE → TI → BI	Indirect	0.086	0.024	3.583	0.043	0.137	0.042	0.137	Partial mediation
	Direct	0.128	0.045	2.844	0.038	0.213	0.040	0.214	
	Total	0.214	0.048	4.458	0.116	0.305	0.118	0.307	
EE → TI → BI	Indirect	0.087	0.023	3.783	0.046	0.138	0.045	0.135	Partial mediation
	Direct	0.113	0.042	2.690	0.029	0.192	0.030	0.193	
	Total	0.200	0.045	4.444	0.112	0.288	0.111	0.288	
FC → TI → BI	Indirect	0.093	0.024	3.875	0.049	0.144	0.049	0.144	Partial mediation
	Direct	0.092	0.045	2.044	0.003	0.179	0.003	0.179	
	Total	0.185	0.046	4.022	0.093	0.273	0.094	0.274	
MC → TI → BI	Indirect	0.042	0.022	1.909	0.002	0.088	0	0.086	Partial mediation
	Direct	0.146	0.043	3.395	0.064	0.228	0.064	0.228	
	Total	0.188	0.044	4.273	0.100	0.275	0.100	0.274	
SI → TI → BI	Indirect	0.112	0.024	4.667	0.069	0.164	0.067	0.162	Partial mediation
	Direct	0.155	0.044	3.523	0.067	0.242	0.066	0.241	
	Total	0.266	0.045	5.911	0.179	0.355	0.178	0.353	

10,000 bootstrap resamples. CI = confidence interval; SE = standard error.

### Experimental results

Inter-rater reliability and rater distributions. Inter-rater reliability was moderate across all expert-rated dimensions (Table 9). Because the inter-rater reliability was only moderate (ICC = 0.635–0.692), the between-group effect-size estimates carry non-trivial measurement error. The reported effect sizes, particularly *d* = 1.30 for visual style, should therefore be interpreted with caution; under reliability correction the underlying effect may be somewhat smaller than the observed estimate. The three raters showed comparable score distributions across dimensions (Supplementary Table S2). Levene’s test indicated no significant variance heterogeneity for any dimension (all *p* > 0.05). Because Welch’s *t*-test does not assume equal variances, it was used for all group comparisons. Expert aesthetic evaluation served as the primary outcome (Table 10). Group A (metacognitive control) significantly outperformed Group B (control) on all three dimensions. For semantic content, Group A (*M* = 5.02, SD = 0.60) scored significantly higher than Group B (*M* = 4.62, SD = 0.58). For visual style, Group A (*M* = 5.13, SD = 0.71) significantly exceeded Group B (*M* = 4.28, SD = 0.59). For spatial composition, Group A (*M* = 4.90, SD = 0.65) was significantly higher than Group B (*M* = 4.52, SD = 0.57). All differences remained significant after Benjamini–Hochberg FDR correction, with the largest gain observed in visual style. As a robustness check (conducted *post hoc*), we additionally tested for batch effects by running a 2 (group) × 2 (session day) ANOVA on the expert-rated outcomes. No significant day × group interaction was found for Semantic content, *F*(1, 76) = 0.42, *p* = 0.519; Visual style, *F*(1, 76) = 0.01, *p* = 0.924; Spatial composition, *F*(1, 76) = 1.06, *p* = 0.308; or the overall score, *F*(1, 76) = 0.76, *p* = 0.386. The main effect of session day was also non-significant on all dimensions (all *p* > 0.05), and group differences remained significant after accounting for session day. These findings provide no evidence that session timing systematically confounded the group differences.

**Table 9:** Inter-rater reliability for expert aesthetic ratings.

Dimension	Cronbach’s α	ICC(2,k)	95% CI
Semantic content	0.691	0.675	[0.537, 0.769]
Visual style	0.695	0.692	[0.556, 0.777]
Spatial composition	0.638	0.635	[0.452, 0.748]

ICC(2,k) = two-way random-effects, absolute-agreement, average-measures intraclass correlation. The 95% CI was estimated using 10,000 bootstrap resamples at the artwork level. Reliability was interpreted as moderate.

**Table 10:** Expert aesthetic evaluation: group comparisons, Levene’s test, and Welch *t*-tests.

Dimension	Group A *M* (*SD*)	Group B *M* (*SD*)	Mean difference [95% CI]	*t*	Cohen’s *d*	Uncorrected P	FDR-adjusted p
Semantic content	5.02 (0.60)	4.62 (0.58)	0.40 [0.14, 0.66]	3.02	0.67	0.003	0.005
Visual style	5.13 (0.71)	4.28 (0.59)	0.85 [0.56, 1.14]	5.81	1.30	<0.001	<0.001
Spatial composition	4.90 (0.65)	4.52 (0.57)	0.38 [0.11, 0.66]	2.80	0.63	0.006	0.006

Group A = metacognitive control group; Group B = control group. M = mean; SD = standard deviation; CI = confidence interval. Levene’s test indicated no significant variance heterogeneity: semantic content, *F*(1, 78) < 0.001, *p* = 1.000; visual style, *F*(1, 78) = 3.72, *p* = 0.057; spatial composition, *F*(1, 78) = 0.61, *p* = 0.439. Because Welch’s *t*-test does not assume equal variances, it was used for all group comparisons. FDR-adjusted *p-*values were corrected using the Benjamini–Hochberg procedure.

The expert and machine-based analyses converged in direction (Table 11). However, the magnitudes of the distance measures are not directly comparable across dimensions because the three dimensions use different feature representations and distance metrics. Both indicated that Group A showed greater within-group heterogeneity than Group B on all three dimensions, and that Group A received higher expert ratings on all three dimensions. Because expert ratings served as the primary outcome, the main conclusion rests on the expert evaluations; the machine-based results provide auxiliary, convergent evidence. These findings provide preliminary experimental evidence that situational metacognitive prompting was linked to higher expert-rated aesthetic quality and lower creative homogeneity within AI-assisted art creation under laboratory conditions.

**Table 11:** Machine-based within-group feature distance results.

Dimension	Group A M distance	Group B M distance	Mean difference (95% CI)	Permutation *p*	FDR-adjusted *p*
Semantic content	0.510	0.276	0.233[0.197, 0.256]	<0.0001	0.00015
Visual style	17.142	9.979	7.163[3.452, 10.814]	<0.0001	0.00015
Spatial composition	4.773	4.271	0.502[0.189, 0.783]	0.001	0.001

Permutation *p-*values were obtained from 10,000 strict permutation tests. FDR-adjusted *p-*values were corrected using the Benjamini–Hochberg procedure. The 95% CIs were estimated using bootstrap resampling with 10,000 replicates. Distances are reported in the native metric of each feature space and are not directly comparable across dimensions. Comparisons are meaningful only within the same dimension.

## Discussion

### Trust as the strongest correlate of behavioral intention

In Study 1, trust in GenAI showed the strongest direct association with behavioral intention (*β* = 0.425, *p* < 0.001). This finding differs from patterns observed in general educational technology research, where performance expectancy is typically regarded as the primary driver of behavioral intention. Students’ willingness to integrate GenAI into their creative processes was associated not only with expected gains in productivity but also, and in this sample more strongly, with perceived trustworthiness (Oc et al., 2024; Rana et al., 2025). The construct as operationalized here is primarily instrumental, supplemented by a modest art-value component. This strong association should not be read as evidence for a fully developed art-value trust dimension.

The role of trust was not uniform across paths in the model. For all five antecedents, the indirect association through trust was significant (bias-corrected 95% CIs excluded zero), and the direct association with behavioral intention remained significant after trust was included. This pattern is consistent with partial mediation for each antecedent. Substantively, partial mediation means that trust is not the sole pathway connecting these antecedents to behavioral intention. Each antecedent is associated with behavioral intention both through trust and through pathways that do not involve trust. For facilitating conditions, the direct association was small but significant (*β* = 0.092, *p* = 0.040; 10,000 bootstrap 95% CI [0.003, 0.179]). Thus, facilitating conditions are associated with behavioral intention partly through trust and partly through a direct association that is independent of trust. This direct association may reflect the possibility that resources, training, and support are associated with behavioral intention not only through their association with trust but also through their association with reduced perceived barriers to use. Because this direct association is close to the significance threshold, the partial mediation pattern should be interpreted cautiously. Overall, the mediation results suggest that trust is an important but not exhaustive explanatory pathway; the antecedents retain direct associations with behavioral intention that are independent of trust.

### Social influence as the dominant correlate of trust

Among the five variables associated with trust, social influence had the highest standardized coefficient (*β* = 0.262, *p* < 0.001), and its indirect association with behavioral intention via trust was also the most pronounced among all indirect paths (indirect coefficient = 0.112), accounting for 42.1% of its total estimated association. In traditional information systems research, social influence is typically understood to operate through normative pressure or imitation mechanisms. However, in artistic creation contexts, its role may be more prominent because social influence may be closely tied to the standards used to evaluate the legitimacy of creative tools and outputs.

Artistic value is not objectively given but is constructed through social negotiation and institutional recognition (Wingström et al., 2024). When teachers, peers, and the broader arts community treat GenAI as a legitimate medium for artistic creation, students may perceive more than just social pressure to conform; they may update their normative beliefs about what constitutes authentic, masterful, and valuable artistic practice (Garcia, 2024; Wu et al., 2026). This socially constructed legitimacy may be closely linked to trust in the technology. The prominent role of social influence is thus consistent with the findings of other scholars’ qualitative studies, which suggest that art students’ attitudes toward AI are deeply embedded in community norms and the process of professional identity formation (Ursavaş et al., 2025). From a theoretical perspective, social influence may be more closely tied to students’ judgments of trust as a cognitive authority, rather than merely serving as a peripheral normative factor in this sample. The remaining three classic dimensions of the UTAUT model were significantly positively associated with trust in GenAI, all meeting the *p* < 0.001 criterion. While the strength of their associations with trust was slightly lower than that of social influence, together they may represent consistent technological and environmental correlates of trust. Educational attainment was significantly positively associated with behavioral intention (*β* = 0.072, *p* = 0.041). This pattern suggests that, in this sample, art students with higher educational attainment reported stronger behavioral intention to use GenAI.

### Metacognitive control as a dual-path correlate of GenAI behavioral intention

In Study 1, metacognitive control was directly associated with behavioral intention (*β* = 0.146, *p* < 0.001) and was also associated with trust (*β* = 0.099, *p* = 0.042); the mediation results were consistent with an indirect association through trust. Taken together, this pattern is consistent with a dual-path process that may be involved in GenAI adoption in artistic contexts. This pattern suggests that metacognitive control may be associated with both greater trust in GenAI and greater willingness to use it.

This finding is consistent with the metacognitive framework for creativity proposed by other scholars, which suggests that metacognitive monitoring and regulation are important for managing the inherent ambiguity and openness of creative tasks (Lebuda and Benedek, 2023). Unlike structured problem-solving tasks with clear termination rules, artistic creation often requires continuous evaluation of whether the output is sufficiently original, aesthetically coherent, and aligned with the creator’s intent (Green et al., 2023). Study 2 results converge with the survey findings on the role of metacognitive control. The pronounced association with visual style suggests that situational metacognitive guidance may be particularly relevant for outputs that deviate from the model’s default stylistic tendencies. However, this should be interpreted as greater output diversity within the feature space, not as direct evidence of enhanced creativity. It should be noted that these effect magnitudes are obtained within a tightly-controlled short-term laboratory setting and may shrink when transferred to authentic classroom contexts. Regarding computational feature-distance outcomes, larger within-group pairwise distances reflect higher heterogeneity among generated images within each experimental group. This metric captures statistical dispersion in semantic embedding, texture-style statistics, and edge-based compositional structure rather than artistic merit. The convergent pattern between expert quality ratings and computational dispersion offers supplementary evidence that metacognitive prompting may be associated with higher expert-rated aesthetic quality and greater output heterogeneity. However, computational diversity alone cannot stand as a proxy for creative quality, and the expert rating scale assesses aesthetic quality rather than creativity itself. Machine-based within-group feature-distance analyses converged in direction: Group A showed greater within-group diversity than Group B across all three dimensions. These findings provide preliminary experimental evidence that, under the conditions examined, metacognitive prompting relative to no prompting is associated with higher expert-rated aesthetic quality and greater output diversity. Theoretically, Study 2 frames situational metacognitive prompting both as a complement to the self-reported individual-difference construct of metacognitive control and as a situational factor amenable to instructional support. This framing carries direct practical implications: because metacognitive prompts can be embedded in educational practice at minimal cost, they may help students engage more actively with GenAI rather than passively accept its outputs. However, Study 2’s conclusions are subject to important boundary conditions. It manipulated situational metacognitive prompts, not the stable construct of metacognitive control; it did not measure trust or test the mediating pathway proposed in Study 1 or the full theoretical model. Moreover, the computational indicators capture specific statistical properties and should not be equated with creativity or aesthetic value; expert ratings therefore serve as the primary evidence. The results are also limited to a short-term task, specific keywords, one image-generation model, fixed parameter settings, and Chinese art students in higher education institutions in Zhejiang Province. Thus, Study 2 should be read as providing complementary and preliminary experimental evidence for one component of the proposed process, not as resolving the GenAI creativity paradox or demonstrating the full mechanism.

### From correlates to practice: cultivating trust and metacognitive control in GenAI-supported art education

Given that Study 1 identifies trust and social influence as strongly associated predictors, arts educators serve as role models who shape perceptions of legitimate artistic practice within studio-based learning contexts (Hamblen, 1985). Social influence in art education extends beyond superficial peer pressure: teachers’ attitudes, feedback styles, and framing of GenAI are associated with students’ professional-identity formation and their value-based judgments about authentic artistic creation. Teacher development programs should prioritize training educators not only in the technical skills required to use GenAI tools but also in teaching strategies designed to help students engage with AI in critical, reflective, and creative ways (Le et al., 2026). Drawing on practice-based pedagogy, the integration of AI-enhanced critique, human-machine collaborative projects, and explicit discussions of authorship and originality can help establish GenAI as a legitimate creative medium while maintaining rigorous artistic standards (Jiang et al., 2026). Such classroom dialogues should address the dimensions of trust related to artistic ethics covered by the scale used in this study—namely, attribution of originality, algorithmic fairness, and respect for creative safety—rather than being limited solely to instrumental performance expectations.

Findings from Study 2 indicate that beyond technical-skill instruction, course designers should integrate metacognitive-control training. Suitable activities include reflective journals documenting collaborative workflows, iterative-focused structured prompt-design tasks, and group discussions on role boundaries. Teaching only prompt-engineering tricks and tool operations cannot fully address risks associated with students passively adopting formulaic algorithm-generated outputs. Curriculum designers may integrate metacognitive-control training into GenAI-infused art courses around the four intervention dimensions: planning, monitoring, regulation, and human-AI role awareness. Practical instructional activities include structured prompt-design worksheets to articulate pre-generation creative goals; iterative-revision logs documenting prompt adjustments based on AI outputs; comparative critique tasks contrasting raw AI outputs with human-refined versions; and post-creation reflective journals that demarcate human creative decisions from algorithmic contributions. For assessment, instructors may assign greater weight to metacognitive process documentation, including creative planning, iterative revisions, and critical evaluation of AI outputs, rather than grading solely on the aesthetic polish of final generated images.

It should be emphasized that these experimental effects were observed within a short-term, controlled laboratory task with fixed keywords and a single image-generation model, which limits the generalizability of results. The metacognitive-prompt template should be treated as a flexible pedagogical scaffold instead of a universal checklist. For open-ended, complex studio assignments in real-world classrooms, educators can adapt and simplify the four-stage prompts according to project demands and student proficiency levels.

## Conclusion and limitations

### Research conclusion

This study explores psychological predictors of art students’ GenAI behavioral intentions within higher art education. Drawing on cross-sectional survey structural equation modeling analysis and controlled laboratory experiments, this paper extends the UTAUT framework to artistic creation contexts. The trust construct operationalized in this research builds upon rather than replaces conventional technology trust. Instead, it builds upon foundational instrumental trust concerning system stability, operational reliability and data security, and further incorporates domain-specific value-related concerns unique to art practice, including perceptions of output originality, authorship attribution, and algorithmic fairness.

Empirical results indicate that trust in the GenAI construct is the strongest direct predictor of students’ behavioral intention to use GenAI for creation. Classic UTAUT antecedents were associated with behavioral intention both directly and indirectly through trust. Among these predictors, social influence correlates most strongly with trust in GenAI. Within artistic contexts, social influence is not limited to normative peer pressure; it corresponds to community- and institution-based evaluations that may inform students’ perceptions of both the technical dependability and artistic-ethical appropriateness of AI-assisted creation.

Metacognitive control shows dual associations with behavioral intention: it is positively associated with behavioral intention directly, and it is also positively associated with trust in GenAI. When learners deploy metacognitive strategies including planning, monitoring, regulation, and clear awareness of human-AI role boundaries, they can better assess system performance and identify potential value-related risks such as copyright ambiguities. Experimental findings show that situational metacognitive support is associated with higher expert-rated aesthetic quality and greater output diversity across semantic content, visual style, and spatial composition.

The present data offer limited preliminary experimental evidence. Even technically stable GenAI systems may yield formulaic outputs under passive user engagement. The current results are consistent with the possibility that metacognitive control may be associated with reduced tendencies toward formulaic outputs, though these observations should be interpreted cautiously given laboratory constraints.

From a practical standpoint, art educators should recognize the dual composition of GenAI-related trust. Instruction should support students to first develop confidence in basic system functions, while also encouraging deliberate reflection on art-specific value considerations such as originality and authorship. The extended UTAUT model integrating instrumental and domain-specific value-based trust provides an empirically grounded reference for subsequent technology-acceptance research in creative education settings.

### Limitations and future directions

Several limitations constrain the interpretation of study findings. First, survey data are cross-sectional. While the experiment identifies effects of situational metacognitive prompts on creative outputs, cross-sectional data cannot establish definitive temporal ordering or causal direction among metacognitive control, trust in GenAI, and behavioral intention.

Second, participants are Chinese art-major students sampled exclusively from Zhejiang Province. Cultural understandings of artistic authorship, institutional AI policies, and baseline expectations for technical-system trust differ across regions and disciplines. Such contextual factors may change the relative weight of instrumental trust versus art-value-aligned trust within the overall trust construct.

Third, the laboratory experiment adopts short-term, constrained creation tasks with fixed keywords and a single GenAI model. Authentic studio assignments feature open-ended themes and long iterative cycles. Observed output-diversity improvements under laboratory conditions may not fully generalize to real-world classroom practice. In addition, computational feature-distance metrics only capture statistical dispersion of image features; they cannot serve as comprehensive proxies for artistic creativity or nuanced subjective value-based trust perceptions.

Fourth, the present design cannot test the GenAI creativity paradox itself. The paradox concerns declining collective-level novelty across independent users of a shared model (Doshi and Hauser, 2024), whereas Study 2 measured within-group output dispersion under a single model. Directly testing the paradox would require multiple groups of participants who share the same model and task, with the collective-level distribution of outputs measured and compared across conditions.

Fifth, a measurement limitation should be acknowledged. Because the two authorship items that formed a separate factor were removed in the EFA, and only two of the five retained items (TI2 and TI4) capture art-specific value concerns, the trust scale used here predominantly measures instrumental technology trust and only partially covers the art-value dimension. Consequently, the present findings should be read as concerning a primarily instrumental trust construct, and a dedicated, validated trust scale for art-specific AI contexts is needed in future research to more fully capture value-laden trust.

Based on these constraints, several directions are suggested for future research. Longitudinal research can track dynamic shifts in instrumental trust, art-specific value-based trust, metacognitive strategies and adoption behaviors as students accumulate ongoing AI co-creation experience. Such designs may help examine whether instrumental trust acts as a precondition for the development of art-oriented value-based trust. Cross-cultural and cross-disciplinary replications are needed to test model external validity. Researchers can explore how cultural and institutional contexts alter the relative salience of instrumental trust and domain-specific value-based trust across fine arts, design, media arts and non-art disciplines.

Follow-up experimental work may further separate and examine the two sub-components of trust. It remains to be tested whether metacognitive interventions exert differential influences on instrumental system trust versus art-specific value-based trust, and whether trust mediates the link between metacognitive prompting and creative outcomes.

Field-based intervention research embedded within real art curricula is also warranted. Curriculum modules may be designed to foster baseline system trust, facilitate critical reflection on art-related value dilemmas, and deliver metacognitive-control training. Further work may develop assessment approaches that balance technical proficiency, documentation of metacognitive processes, and critical reflection on authorship and algorithm-related ethical issues. Mixed-methods research combining quantitative scales and qualitative interviews can advance understanding of how art students navigate tensions between tool performance and artistic-value considerations during human-AI collaborative creation.

## Data Availability

The datasets presented in this article are not readily available because data access is provided only upon reasonable request to the authors and will not be released without such a request. The dataset can only be used for academic research purposes. Access approval is required from the responsible ethics committee. Data sharing must comply with applicable privacy regulations. Before sharing, all datasets will undergo de-identification to remove identifying personal information, as the original data. Requests to access the datasets should be directed to Renhao Zhu, 519109702@qq.com.
