# Supplementary material for: Supporting creative agency: metacognitive control and trust in generative AI art education
Source: Front Psychol. 2026 Oct 29;17:1997631. doi: 10.3389/fpsyg.2026.1997631 (PMC13755252; doi:10.3389/fpsyg.2026.1997631)
Supplement: Supplementary file 1 [file Table_1.docx]

**Supplementary Documents**

**Table S1**

*Four-Stage Metacognitive Control Prompts*

| **Stage** | **Dimension** | **Guiding prompts (answer in writing before continuing)** |
| --- | --- | --- |
| Stage 1 | Planning | 1. What is my creative intent for this image? What emotion, story, or idea do I want to convey?  2. What semantic content (subject, setting, action, mood) will I specify?  3. What visual style do I aim for (e.g., realistic, illustrative, cinematic, minimalist, fantastical)?  4.How do I want the spatial composition arranged (framing, perspective, focus, balance)?  5. What concrete details in my prompt can steer the AI toward my vision rather than its default output? |
| Stage 2 | Monitoring | 1. Does the current AI output align with my creative direction and aesthetic goals?  2. Which aspects (semantic content, visual style, spatial composition) meet expectations, and which do not?  3. Am I consciously evaluating the output, or merely accepting whatever the AI generates?  4. Have I noticed any gap between my intent and the machine's interpretation? |
| Stage 3 | Regulation | 1. Based on the output feedback, what should I change in my prompt?  Should I revise keywords, add style or composition cues, or adjust the level of detail?  Should I try a different angle, framing, color tone, or stylistic direction?  Which ideas should I keep, develop, refine, or discard to get closer to my artistic goal? |
| Stage 4 | Self-Reflection & Role Awareness | Does the final image reflect my own creative intent, or merely the AI's default result?  What were my role and the AI's role in this creation? Who made the key creative decisions?  What did I learn about my own prompting and decision-making strategies?  If I did it again, what would I do differently? |

**Table S2**

*Rater-level score distributions*

| **Dimension** | **Rater** | **M** | **SD** | **Median** | **IQR** | **Min** | **Max** |
| --- | --- | --- | --- | --- | --- | --- | --- |
| Semantic content | E1 | 4.61 | 0.75 | 5.0 | 1.0 | 3 | 6 |
|  | E2 | 4.95 | 0.78 | 5.0 | 1.0 | 3 | 7 |
|  | E3 | 4.89 | 0.84 | 5.0 | 1.0 | 3 | 7 |
| Visual style | E1 | 4.58 | 1.09 | 4.5 | 1.0 | 3 | 7 |
|  | E2 | 4.81 | 0.96 | 5.0 | 1.0 | 2 | 7 |
|  | E3 | 4.74 | 0.91 | 5.0 | 1.0 | 2 | 7 |
| Spatial composition | E1 | 4.60 | 0.77 | 4.5 | 1.0 | 3 | 6 |
|  | E2 | 4.80 | 0.88 | 5.0 | 1.0 | 3 | 7 |
|  | E3 | 4.73 | 0.86 | 5.0 | 1.0 | 3 | 6 |

Note. E1–E3 = three blinded expert raters. IQR = interquartile range.

**Table S3**

*EFA results for the trust scale with and without authorship items*

| **Item** | **Factor 1 loading** | **Factor 2 loading** |
| --- | --- | --- |
| TI1 | .716 | — |
| TI2 | .714 | — |
| TI3 | .724 | — |
| TI4 | .788 | — |
| TI5 | .759 | — |
| BHA1 (authorship) | — | .773 |
| BHA3 (authorship) | — | .845 |
| Eigenvalue | 2.983 | 1.213 |
| Variance explained (%) | 42.61 | 17.33 |
| Cumulative variance (%) | 42.61 | 59.94 |
| Cronbach’s α (7 items) | .769 |  |
| Cronbach’s α (5 items) | .803 |  |

*Note*. EFA with principal axis factoring and varimax rotation. Loadings below .40 are not shown. The two authorship items formed a separate factor and were removed before CFA.

**Table S4**

*Nested model comparison for discriminant validity between TI and BI*

| **Model** | **χ²** | **df** | **CFI** | **TLI** | **RMSEA** | **Δχ² (Δdf)** | **p** |
| --- | --- | --- | --- | --- | --- | --- | --- |
| Two-factor model (TI, BI) | 22.90 | 26 | 1.000 | 1.002 | .000 |  |  |
| One-factor model (TI+BI merged) | 2035.07 | 28 | .249 | .035 | .400 | 2012.17 (2) | < .001 |

Note. The one-factor model constrained all TI and BI items to load on a single factor. The two-factor model allowed TI and BI to correlate freely. ΔCFI > .01 and ΔRMSEA > .015 indicate a meaningful difference in fit.

**Expert Review Form**

— Experiment on Generative-AI-Assisted Art Creation (Study 2) —

**I. Study Information**

Study Title: Effects of Metacognitive Control on the Diversity of Generative-AI-Assisted Art Creation (Study 2)

Research Institution: Hengdian Film and Television College

Researcher: Zhu Renhao (Phone number and email address are shown in Part VIII)

Study Purpose: Invite expert reviewers to independently score 80 digital artworks created by two groups of participants (Group A: Metacognitive Control Group; Group B: Control Group) who used generative-AI tools. Scoring covers three dimensions: semantic content, visual style and spatial composition, so as to compare differences between artworks of the two groups in the above-mentioned dimensions.

Ethics Review: ☐ This study has passed ethics review (Approval No.: __________)

**II. Reviewer Information (Please fill in)**

| **Name** |  | **Institution** |  |
| --- | --- | --- | --- |
| **Phone Number** |  | **Professional Title** |  |
| **E-mail** |  | **Review Date** |  |

**III. Review Instructions**

(1) Review Subjects: A total of 80 digital artworks, numbered A01–A40 and B01–B40. Artworks are displayed one by one by number in the separately provided Portfolio. Please complete scoring against artwork numbers.

(2) Review Task: Assign an integer score ranging from 1 to 7 for each artwork across the three dimensions of semantic content, visual style and spatial composition (1 = lowest degree; 7 = highest degree). Scoring criteria are detailed in Part IV.

(3) Review Requirements: Please finish the review independently and do not discuss scoring results with others. Scoring shall be based on actual observation of artworks; do not make any inferences regarding creator identity, creation process or generation method. Score each artwork sequentially to avoid missing or duplicate scoring.

(4) If an artwork cannot be assessed, mark “Cannot be judged” in its scoring field and state the reason. Do not arbitrarily assign intermediate scores.

**IV. Scoring Criteria (1–7-point Anchors for Three Dimensions)**

**Semantic Content (Semantic Richness and Originality of Artworks)**

| **Score** | **Descriptive Definition** |
| --- | --- |
| 1 | Vacant content, absent theme, obvious copy of common templates or simple repetition of elements |
| 2 | Poor content, ambiguous theme, monotonous elements |
| 3 | Mediocre content, recognizable yet plain theme, lack of novelty |
| 4 | Fairly standard content, clear theme with a small amount of personal expression |
| 5 | Relatively rich content, distinct theme with novel elements or combinations |
| 6 | Rich and layered content, profound theme, strong originality |
| 7 | Extremely rich and unique content, distinct and profound theme, outstanding originality |

**Visual Style (Identifiability, Consistency and Expressiveness of Style)**

| **Score** | **Descriptive Definition** |
| --- | --- |
| 1 | No definite style, chaotic picture, or direct replication of common generative-AI styles |
| 2 | Crude single style, low expressiveness |
| 3 | Recognizable yet conventional style, average expressiveness |
| 4 | Clear and unified style with moderate expressiveness |
| 5 | Distinct style, strong expressiveness, presenting personalized visual language |
| 6 | Unique and coherent style, powerful visual impact |
| 7 | Highly original style, mature visual language and extremely high recognizability |

**Spatial Composition (Balance, Hierarchy and Originality of Composition)**

| **Score** | **Descriptive Definition** |
| --- | --- |
| 1 | Random composition, unbalanced or absent main subject |
| 2 | Simple composition, over-centered or over-symmetrical layout, lack of layers |
| 3 | Reasonable yet formulaic composition, lack of variation |
| 4 | Balanced composition with basic primary-secondary relationship and layers |
| 5 | Well-considered composition, distinct layers, moderately innovative perspective |
| 6 | Exquisite composition, innovative perspective, expressive spatial relations |
| 7 | Highly creative composition, unique spatial utilization and excellent visual narration |

**V. Artwork Scoring Sheet**

Please assign an integer score of 1–7 for each artwork in the three respective dimensions.

| **Artwork No.** | **Semantic Content (1–7)** | **Visual Style (1–7)** | **Spatial Composition (1–7)** |
| --- | --- | --- | --- |
| A01 |  |  |  |
| A02 |  |  |  |
| A03 |  |  |  |
| A04 |  |  |  |
| A05 |  |  |  |
| A06 |  |  |  |
| A07 |  |  |  |
| A08 |  |  |  |
| A09 |  |  |  |
| A10 |  |  |  |
| A11 |  |  |  |
| A12 |  |  |  |
| A13 |  |  |  |
| A14 |  |  |  |
| A15 |  |  |  |
| A16 |  |  |  |
| A17 |  |  |  |
| A18 |  |  |  |
| A19 |  |  |  |
| A20 |  |  |  |
| A21 |  |  |  |
| A22 |  |  |  |
| A23 |  |  |  |
| A24 |  |  |  |
| A25 |  |  |  |
| A26 |  |  |  |
| A27 |  |  |  |
| A28 |  |  |  |
| A29 |  |  |  |
| A30 |  |  |  |
| A31 |  |  |  |
| A32 |  |  |  |
| A33 |  |  |  |
| A34 |  |  |  |
| A35 |  |  |  |
| A36 |  |  |  |
| A37 |  |  |  |
| A38 |  |  |  |
| A39 |  |  |  |
| A40 |  |  |  |
| B01 |  |  |  |
| B02 |  |  |  |
| B03 |  |  |  |
| B04 |  |  |  |
| B05 |  |  |  |
| B06 |  |  |  |
| B07 |  |  |  |
| B08 |  |  |  |
| B09 |  |  |  |
| B10 |  |  |  |
| B11 |  |  |  |
| B12 |  |  |  |
| B13 |  |  |  |
| B14 |  |  |  |
| B15 |  |  |  |
| B16 |  |  |  |
| B17 |  |  |  |
| B18 |  |  |  |
| B19 |  |  |  |
| B20 |  |  |  |
| B21 |  |  |  |
| B22 |  |  |  |
| B23 |  |  |  |
| B24 |  |  |  |
| B25 |  |  |  |
| B26 |  |  |  |
| B27 |  |  |  |
| B28 |  |  |  |
| B29 |  |  |  |
| B30 |  |  |  |
| B31 |  |  |  |
| B32 |  |  |  |
| B33 |  |  |  |
| B34 |  |  |  |
| B35 |  |  |  |
| B36 |  |  |  |
| B37 |  |  |  |
| B38 |  |  |  |
| B39 |  |  |  |
| B40 |  |  |  |

Reviewer Signature: ____________________	Date: ____________________

**VI. Independent Review Statement by Expert Reviewer**

(1) I have fully read and understood the scoring criteria and operational definitions in Part IV of this form.

(2) I have completed scoring for all artworks independently, without influence from any other person and without discussing scoring results with others.

(3) I have no conflict of interest with this study, the research team or the artwork creators.

(4) I promise that scoring is based on direct observation of artworks, without incorporating inferences about creator identity, creation process or generation method.

(5) I consent that scoring data can be used for data analysis of this study. I am aware that data will be anonymized and only presented in aggregated statistical form.

Reviewer Signature: ____________________	Date: ____________________

**VII. Personal Information and Privacy Protection Statement**

In accordance with the Personal Information Protection Law of the People's Republic of China (Adopted at the 30th meeting of the Standing Committee of the 13th National People's Congress on August 20, 2021; effective November 1, 2021), this study hereby informs you of personal-information-processing matters involved in the review:

(1) Collected Information: Name, institution and professional title, phone number, e-mail address, review date.

(2) Collection Purposes: Reviewer qualification verification, communication during review, attribution management of scoring data, and acknowledgement in research outputs (if applicable).

(3) Processing Methods: The above-mentioned information shall only be used by the research team for purposes specified in Item 2. No information shall be provided to any third party or used for any other purposes. Scoring data and personal identifiable information shall be stored separately. No personally-identifiable form will appear in research reports or papers.

(4) Security Measures: Personal information will be encrypted and stored with designated custodians. Upon study completion, data will be deleted or anonymized within the agreed time frame.

(5) Your Rights: You have the right to access, copy, correct, supplement or delete your personal information, and the right to withdraw consent. Withdrawal of consent shall not affect processing activities lawfully conducted prior to withdrawal.

(6) Inquiries and Contact: If you intend to exercise the above-mentioned rights or have any questions, please contact the researcher (contact information in Part VIII).

Informed Consent: ☐ I have fully read the above content and consent to the collection and use of my personal information by this study as specified above.

Reviewer Signature: ____________________	Date: ____________________
